# Parental educational homogamy and under-five mortality in sub-Saharan Africa: Clarifying the association’s intricacy

**DOI:** 10.1016/j.sciaf.2019.e00255

**Published:** 2019-12-19

**Authors:** Ayo Stephen Adebowale, Adeniyi Francis Fagbamigbe, Oyewale Morakinyo, Taiwo Obembe, Rotimi Felix Afolabi, Martin Enoch Palamuleni

**Affiliations:** a Department of Epidemiology and Medical Statistics, University of Ibadan, Ibadan, Nigeria; b Department of Environmental Health Sciences, University of Ibadan, Ibadan, Nigeria; c Department of Health Policy and Management, University of Ibadan, Ibadan, Nigeria; d Population Training and Research Unit, North-West University, Mafikeng, South Africa

**Keywords:** Under-five survival, Homogamy, Parental education, Sub-Saharan Africa

## Abstract

Worldwide, under-five mortality (U5M) rate is highest in sub-Saharan Africa (SSA). There exists a gap in knowledge on the pathway through which Parental Educational Homogamy (PEH) influences U5M in SSA. In this study, we tested the research hypothesis’ PEH is not associated with under-five children’s survival probability in SSA.

Demographic and health survey datasets for 21 SSA countries were analyzed. Cross-sectional design and multi-stage cluster sampling technique were used for sample selection in each of the countries under investigation. The dependent variable was the survival status of a newborn to age 59 months while the main independent variable was PEH generated from information on wife’s and husband’s level of education. Data were analyzed using Chi-square test, Cox-proportional hazard model and Brass-adjusted model (*α*=0.05).

Under-five mortality rate ranges from 56/1,000 live born in South Africa to 190/1,000 live born in Sierra-Leone. Across countries, U5M rate was higher among the children of parents with at most primary education than that of parents who had at least secondary education. This pattern of U5M rate was also observed for children of parents where husbands were more educated than their wives. Maternal age at birth, sex of the child, toilet facility, type of cooking fuel, tetanus injection during pregnancy, and birth weight were significantly associated with U5M in 14, 11, 8, 7, 11, 14 and 20 countries respectively. A significant relationship was established between PEH and U5M in 11 of the 21 studied countries but was identified as a predictor of U5M in Congo Democratic Republic, Gambia and Zimbabwe.

Parental educational homogamy exhibits a pattern of relationship with U5M in SSA. Ensuring that individuals particularly women have at least secondary education before childbearing will facilitate an U5M reduction in SSA.

## Introduction

Under-five Mortality (U5M) rate defined as the probability of dying between age 0 and 5 years is an important health indicator and index of development in all nations. Globally, about 5.5 million children under five years old died in 2017 (93 deaths per 1000 live births) compared to 12.6 million recorded in 1990 (39 deaths per 1000 live births) [1]. Despite this marked global reduction in U5M rate in the past few decades, there remains a huge gap between high-income (5 deaths per 1000 live births) and low-income (69 deaths per 1000 live births) countries [1]. Under-five mortality rate in developed regions like Northern America, Europe, Japan and Australia/New Zealand ranges from 5.3 to 6.5 death per 1000 live birth [1]. In South Asia, Latin America/Caribbean, and Northern Africa, the U5M rate was 44.8, 17.8, and 31.4 per 1000 live birth respectively [1,2]. Compared to other world regions, sub-Saharan Africa (SSA) has the highest U5M rate (75.5 per 1000 live birth) [1,2]. The likelihood that children in SSA will die before the age of 5 years is more than 15 times than children in high-income countries [3]. The consistent high U5M rate in SSA remains a threat to national development across countries in the region and underscores the need to come up with measures to curb the trend.

The Sustainable Development Goals (SDGs) emphasized the need to reduce U5M rate to not more than 25 per 1000 live births [4] by the year 2030. It is doubtful if this target would be met by some countries in SSA if the current trend in U5M rate is sustained. In SSA, U5M is mostly attributed to preventable or treatable causes like complications during birth, pneumonia, diarrhea, neonatal sepsis and malaria [5]. Thus, there is an urgent need to address the underline factors through which these U5M causative mechanisms are rooted in SSA. The relationship between sociocultural, health, environmental factors and U5M has been adequately explored in the literature [6-8]. However, there is a gap in knowledge particularly in SSA about how parental educational homogamy (PEH) influences U5M.

Homogamy is a marriage between individuals who are, in some culturally-important way, similar to each other [9] and often referred to as assortative mating. The choice of partner for marriage is at times based on the individuals match on the level of formal education attained or other characteristics. Becker in his theory of marriage propounded that “likes marry likes when the characteristic is complementary but not when it is substitutable” [10]. In most situations, the reason why two individuals with the same level of education often get married is that they tend to appreciate the same public goods or leisure.

Education is likely to be complementary in consumption and decision orientation of spouses at the family level. Educational homogamy, therefore is the tendency of men and women to marry partner who has acquired a similar level of education to theirs. Earlier studies have shown an increase in educational homogamy [11-13]. Parental educational homogamy in the context of this study is defined as the combination of the spousal’s educational level. In the traditional African Society, the belief is that women’s activities are limited to the provision of care for her immediate family. In this perspective, most women have no formal education and parents were the ones who marry wife for their sons. In contemporary times, the literacy level has increased among women and almost at pal with that of men [14]. Parents barely have control over their children in terms of decision on who to marry because children spend more time at school and outside their immediate family environment. Therefore, education has become central to both the cultural perception of partnership and socioeconomic status in Africa.

The extent to which spouses are similar on traits like educational attainment may be an important component of inequality among families [15]. Given the significance of educational homogamy, a key question that remains unanswered in SSA is how PEH is related to U5M across countries in the region. Good education may attract better income and has been shown to define the family’s wealth status in many societies [16,17]. Education of at least secondary level can equip couple with the knowledge and skills they need to be competitive in the workforce, helping them to earn more and stimulating sustained economic growth and self-reliance. Good education also has transformative health and economic advantages for family members and has been previously linked to lowering U5M in many countries [18-20]. In this study, we tested the research hypothesis’ PEH is not associated with under-five children’s survival probability in SSA. Therefore, we aimed at determining the level of U5M across the categories of PEH. We also examined the relationship between PEH and U5M while controlling for other factors across selected countries in SSA. The study provides information that could be useful to assist policy makers on their quest for reasons for high U5M in SSA.

## Method

### Study area

**Fig. 1.**
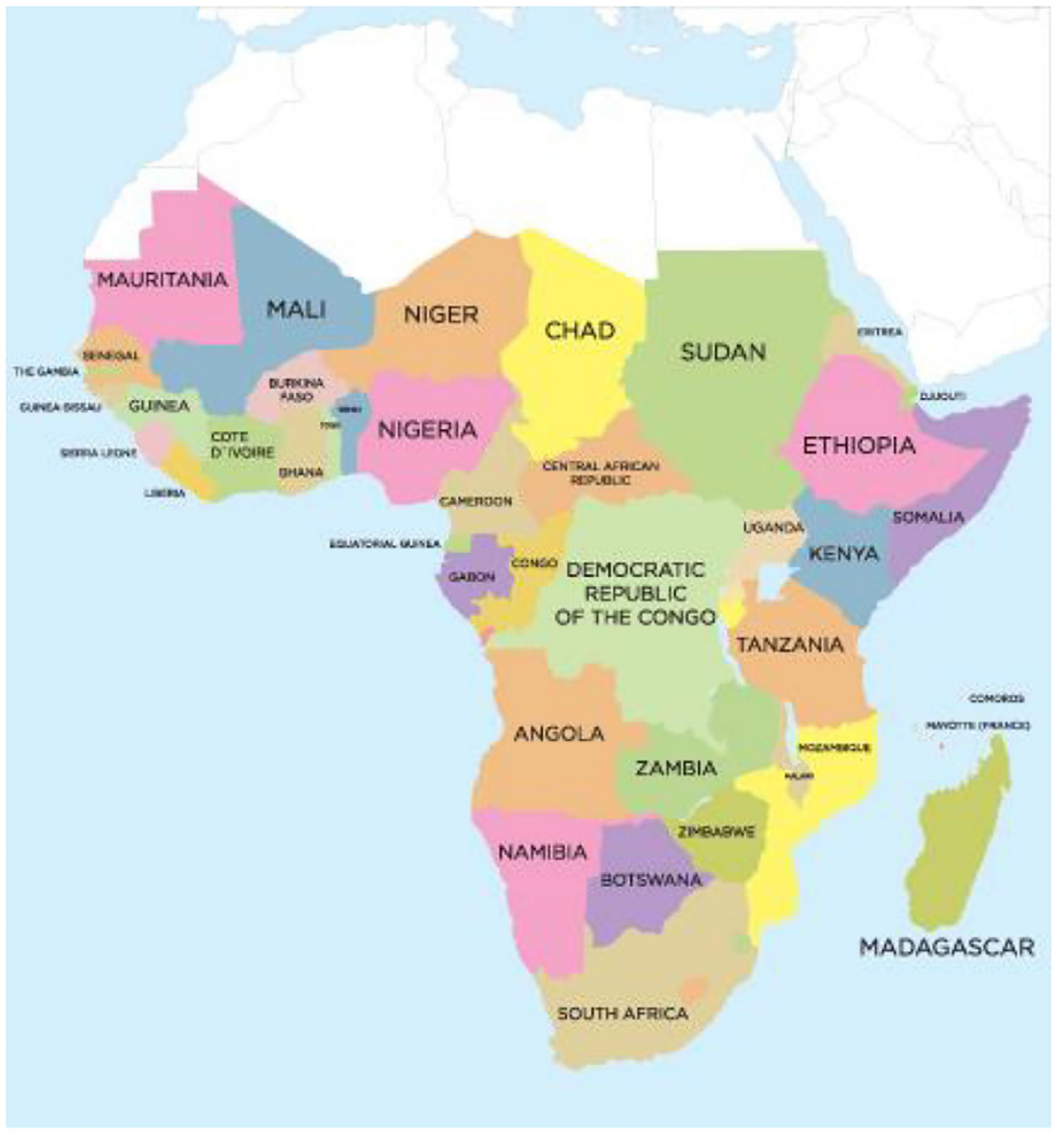
Map of sub-Saharan Africa.

Sub-Saharan Africa is a sub-region in Africa (Fig. 1) and it consists 46 countries out of the 54 countries in Africa. The total population of the region was about 1.049 billion in 2018 and this population is evenly distributed by sex [21]. The countries in the region are characterized by high childhood mortality [21]. Statistics showed that the literacy rate for sub-Saharan Africa was 65% in 2017, an implication that one-third of the people aged ≥15 years were unable to read and write [14]. In the past, men are sent to school to acquire western education while women are confined to domestic works. However, in recent times, there is a paradigm shift in this arrangement as the literacy level of women has increased, with variation existing between and within countries in the region [14]. The people in the region are diverse in culture with each having their own beliefs and traditions.

### Data collection and sampling

In this study, demographic health survey datasets were analyzed for 21 purposefully selected countries in sub-Saharan Africa. The selection was based on data availability and the survey year (not earlier than 5-years period prior the data analysis for this study). Consequently, 6 countries were selected from West Africa (Nigeria, Liberia, Ghana, Senegal, Sierra-Leone, Gambia), 9 from the East Africa (Burundi, Ethiopia, Uganda, Kenya, Malawi, Tanzania, Rwanda, Zambia, Zimbabwe), while 3 were selected from Central (Angola, Congo Democratic Republic) and Southern Africa (Lesotho, Namibia, South Africa).

The data collection method (cross-sectional and cluster design) for all the included countries is similar and from the same source [22]. A two-stage sampling technique was used for the sample selection [22]. The Demographic and Health Surveys (DHS) was basically designed to cover 100% of the target population in each country. The target population for this study are women aged 15–49 years, their children under five years of age and spouse living together in residential households. Four main questionnaires were used to collect the data, but the data analysis for this study was based on the woman’s questionnaire, which has questions about the woman, birth history of all children ever born (alive or dead), including the child’s sex, date of birth, age, and survival status. The birth history was the basis for selecting children under certain ages for the maternal and child health sections of the questionnaire.

The general sampling principles across the countries are the same. In some countries, exclusion of some areas may be essential because of inaccessibility, violence or instability, nevertheless these issues were considered at the planning stage of the survey. In DHS, countries often rely on an existing sampling frame, an existing master sample, or a sample of a previously executed survey of sufficiently large sample size, for the selection of subsamples of desired size. The common frame used in DHS is the list of Enumeration Areas (EAs) from the most recently completed population census in each country. A complete list of EAs which covers the survey area entirely is mostly used sampling frame. Comprehensive information about sample selection in each country included in this study is available to explore by the readers on the web-platform of the data originator.

The original sample selected for each country based on child’s record file which forms the basis of analysis in this study was Nigeria (31,482), Liberia (7606), Ghana (5884), Senegal (12,185), Sierra-Leone (11,938), Gambia (8088)), Burundi (13,192), Ethiopia (10,641), Uganda (15,522), Kenya (20,964), Malawi (17,286), Tanzania (10,233), Rwanda (7856), Zambia (13,457), Zimbabwe (6132), Angola (14,322), Chad (18,523), Congo Democratic Republic (18,716) and Lesotho (3138), Namibia (5046), South Africa (3548). However, in the current study, analyses were based on subjects with complete information on; childhood survival status, educational attainment of both husband and wife. Multiple births and mothers who are either separated or widowed or not cohabiting or not in marital union were excluded from the analysis.

### Variable description

The dependent variable was child survival status within the first 59 months of life. This was categorised as; alive or dead. The PEH was the main independent variable and was generated from the information on level of education of wife and that of their husband. Thus, four categories were obtained as follows;

PEH={1=bothcouplehaveatmostprimarylevelofeducation2=husbandhasatmostprimary&wifehasatleastsecondarylevelofeducation3=husbandhasatleastsecondary&wifehasatmostprimarylevelofeducation4=bothcoupleshaveatleastsecondarylevelofeducation}

Individuals with no formal education and primary education were merged because previous studies have shown little or no difference in U5M rate among parents with no formal and primary education [23,24]. Other independent variables used in this study are as follows;

**Table:** 

Socio-demographic	Child’s biodata	Health-related	Environmental
Maternal age at child birth Children ever born Residence Religion Household wealth	Sex Birth order Birth weight	Tetanus injections before birth ANC visit Place of Delivery Preceding birth interval	Source of drinking water Toilet facility Type of cooking fuel

The selection of the independent variables for analysis was based on Mosely and Chen [25] and Garenne and Gakusi [26] frameworks for childhood mortality determinants. However, some of the variables in the frameworks were not included in the current study mainly because either they were not included during the survey or dropped to avoid multi-collinearity or not adequately captured in the data. The categorisation of the variables like type of source of drinking water, toilet type and cooking fuel type was in accordance with the recommendation of the data originator and inline what was earlier reported [7,22].

### Data analysis

The extracted data were weighted before use for descriptive analysis but unweighted at the level of multivariate analysis. Data were analyzed using Chi-square test, Cox-proportional hazard model. In order to further clarify the pattern of the relationship between PEH and U5M, demographic model was used to estimate U5M rate in each of the countries across the four categories of PEH. The Chi-square test was used to examine the association between the PEH and U5M. Other independent variables were also involved in the test. To facilitate selection of variables into the multivariate model, unadjusted hazard ratio (Cox-proportional hazard model) of U5M was generated for each of the independent variable. However, variables that were found to be statistically significant (*α* =0.10) at this level of analysis were used in the multivariate analysis as adjustment mechanism for the relationship between PEH and U5M (*α* =0.05).

### Multivariate analysis

In the Cox-proportional hazard model, the status variable is the survival status of the child after delivery with the main indicator as death, the time to event variable is the number of months the child spent in the study. A common feature of child survival data sets is that they contain censored observations. Censored data arises when a child’s life length is known to occur only in a certain period of time. Thus, children who died at any time before age 59 months after birth are the cases, those who were alive for at least the first 59 months of life and those where information on survival status cannot be ascertained at the end of 59th month are said to be censored.

A Cox-proportional hazard model is a form of survival analysis technique. The basic quantity fundamental in survival analysis is the hazard function. This function is the hazard rate. The hazard rate is defined by

$$h(x)=\underset{\Delta x\to 0}{\text{lim}}\frac{P(x\leq X<x+\Delta x∕X\geq x)}{\Delta x}$$

Therefore,

$$h(x)=f(x)∕S(x)=-dlog_{e}[S(x)]∕dx$$

A related quantity is the cumulative hazard function H(x) given by;

$$H(x)=\int_{0}^{x}h(u)du=-log_{e}[S(x)]$$

Thus,

$$S(x)=exp[-H(x)]=exp\left[-\int_{0\;\;}^{\;x}h(u)du\right]$$

Where *h*(*u*)Δ*x* is the probability of a individual child of age *x* experiencing mortality in the next instant. This function was used in determining the appropriate death distributions utilizing other information about the mechanism of death and for describing the way in which the chance of experiencing mortality changes with time. The general proportional hazards model is,

$$h_{i}(t)∕h_{0}(t)=e^{\beta_{1}x_{1i}+\beta_{2}x_{2i}+\ldots +\beta_{p}x_{pi}}$$

Thus it is a linear model for the logarithm of the hazard ratio (*e^β_i_^*) and there is no assumption concerning the distribution of *h*_0_ (*t*), the baseline hazard function. Thus the model is non-parametric with respect to time but parametric in terms of the exploratory variables *x*_1*i*_, *x*_2*i*_, … ,*x*_*pi*_ and is referred to as semi-parametric model. The coefficients *β*_1_, *β*_*2*_, … ,*β*_*p*_ of the equation are regression parameters and their interpretation depends on the signs of their magnitude. A positive sign means that the hazard ratio of U5M is higher and thus the prognosis worse in that category relative to a reference category and the reverse is the case if the sign negative. However, a coefficient that fails to attain statistical significance (*p*<0.05) does not contribute significantly to the variability of the U5M.

Cox proportional hazards model has several assumptions, but the most important among them is the proportional hazards assumption [27]. This assumption was checked using the scaled Schoenfeld residuals. The plot shows a random pattern against time, an evidence that the proportional hazard assumption is satisfied. This was supported by a non-significant relationship between residuals and time. A further plot of the graph of the log(−log(survival)) against log of survival time graph resulted in parallel lines, an indication of proportional hazard.

### Estimation of under-five mortality rate

The Brass demographic technique as modified in tool for demographic estimation [28,29] was used to estimate U5M rate across the spectrum of PEH in each country. The technique is an indirect method for the estimation of childhood mortality in countries with deficiencies in vital registration system as the case in most SSA countries. The estimate was based on information obtained from women on children ever born and children surviving classified by 5-year age group. The classification was used to estimate the probability of dying from to exact age (x) as;

q(x)=k(x)×D(x)

where D(x) is the number of dead children in each age group and;

$$k(x)=a(i)+b(i)(P_{1}∕P_{2})+c(i)(P_{2}∕P_{3})$$

Regression equations which relate the multipliers k(x) to indices of fertility schedule were fitted (). The estimate of probability of surviving obtained from this method was further adjusted using Brass 1-parameter model *Y* = *α* + *β*Y^s^ (n) i.e. logit{l(x)} = ∝ + *β*logit{l^s^(x)}. The estimated probabilities of dying by exact childhood age x, _5_q_x_, were converted into a value of *α*, which is the level parameter of a system of relational logit model life tables. The *α* was used to estimate the corresponding probability of dying between age 0 and 5, _5_q_0_ :

$$\alpha =\frac{1}{2}ln\left(\frac{{\;}_{n}q_{0}}{1-{\;}_{n}q_{0}}\right)-Y^{s}(n)$$

where the estimates of _5_q_0_ and the *Y*^*s*^ (*n*) values are logit transformations of the standard life table. The west class of the Coale-Demeny model life table was used as the standard. Then for each *α*, _5_q_0_ was estimated using the equation:

$${\;}_{{}_{5}}{\hat{q}}_{0}=\frac{e^{2(\alpha +Y^{s}(n))}}{1+e^{2(\alpha +Y^{s}(n))}}$$

## Results

**Fig. 2.**
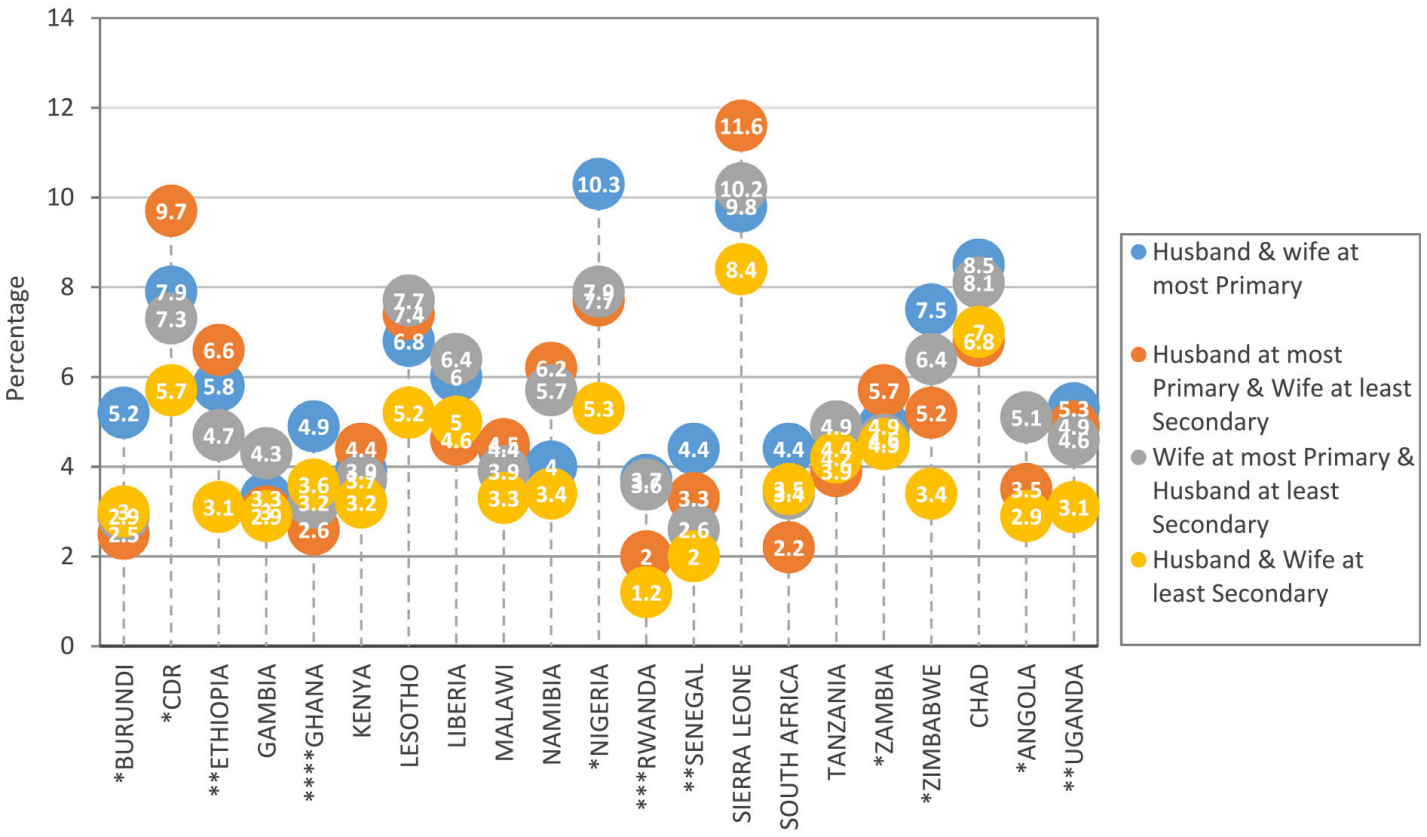
Percentage Distribution of Under-five Mortality by Country according to Educational Homogamy.

Fig. 2 shows the percentage of deaths among under-five children across different classes of PEH in each of the studied countries in SSA. A significant association was established between PEH and U5M in countries like Burundi, Congo Democratic Republic, Ethiopia, Ghana, Nigeria and Rwanda. Others include Senegal, Uganda, Zimbabwe and Angola. Percentage of under-five deaths was lowest in families where both husband and wife have at least secondary education in 15 out of the 21 countries studied. In Uganda for instance, 3.1% of under-five deaths were experienced among couples where both husband and wife have at least secondary education compared with 5.3% under-five deaths being experienced by their counterparts who have at most primary education (*p* = 0.001). The difference in percentage of under-five deaths among recent live births between marital union where husband and wife have at most primary education (10.3%) and those where the spouse has at least secondary education (5.3%) was mostly striking in Nigeria (*p*<0.001). In 14 of the countries, the percentage of the live born that resulted to death before the age of 5 years was lower in households where the wife is more educated than husbands compared to where the husband has acquired more education than the wife. This is the case for countries like Ghana (2.6% vs 3.2%), Rwanda (2.0% vs 3.6%), Angola (3.5% vs 5.1%), Burundi (2.5% vs 2.9%), Gambia (3.0% vs 4.3%), Liberia (4.6% vs 6.4%), South Africa (2.2% vs 3.4%), Zimbabwe (5.2% vs 6.4%) and others. The data further showed that countries in West Africa (Sierra-Leone and Nigeria) are more affected by under-five deaths, closely followed by Central African countries (Chad and Congo Democratic Republic).

The distribution of under-five deaths according to child, parental and some health related characteristics is presented in Table 1. A significant association between parental place of residence and under-five mortality was established in 6 (Angola, Ethiopia, Malawi, Nigeria, Uganda and Zimbabwe) out of the 21 countries. Consistently, higher U5M was reported in rural areas than the urban areas across these 6 countries. In Nigeria, U5M occurred in 9.7% of live born children in rural areas compared to 5.9% observed in the urban areas. Maternal age at the birth of the child under investigation was found to be significantly associated with U5M in 14 countries and the usual U-shape pattern of death existed in the association. Under-five deaths were mostly experienced by women at the extreme age groups (<20 years and ≥35 years). In Angola, under-five death was 3.9% among women who had their children from age 25 to 34 years and 6.2% among those who have theirs in ages of at least 35 years. In Tanzania, the proportion of live born that resulted to death before the child attains age 5 years was 6.6%, 3.6%, 3.7% and 5.3% among mothers who had their birth at age <20 years, 20–24 years, 25–34 years and ≥35 years respectively.

The number of children previously born to a woman was found to be significantly associated with U5M in sixteen countries. There was evidence of higher U5M among women of parity six and above than those in lower parity and this pattern exists in almost all the countries. There was sex difference in U5M with the percentage of death among live born children before reaching the age of five years being higher among males than females. Toilet facility, type of cooking fuel, size of the child at birth and birth weight were significantly associated with U5M in some of the countries. In Burundi and Ethiopia for instance, U5M was 4.9% and 5.9% in households where unimproved toilet facilities are being used compared to 2.3% and 3.3% observed in households using improved toilet facilities respectively. The U5M was significantly higher among children living in households where the type of cooking fuel commonly used was biomass [Nigeria (9.2%), Namibia (5.5%), Zimbabwe (4.9%)] than those who use clean fuel [Nigeria (5.1%), Namibia (2.8%), Zimbabwe (2.8%)]. As expected, the burden of U5M was higher among under-five children who were underweight (weigh less than 2.5 kg) at birth than those who had either normal weight (2.5kg-3.49 kg) or overweight (≥3.5 kg) at birth. It is important to note that birth weight was significantly associated with U5M in 20 out of the 21 countries being studied. Variables such as preceding birth Interval, whether tetanus injection was given during pregnancy taken status and place of delivery were all associated with U5M in Congo Democratic Republic, Ethiopia, Kenya, Nigeria and Zimbabwe.

**Fig. 3.**
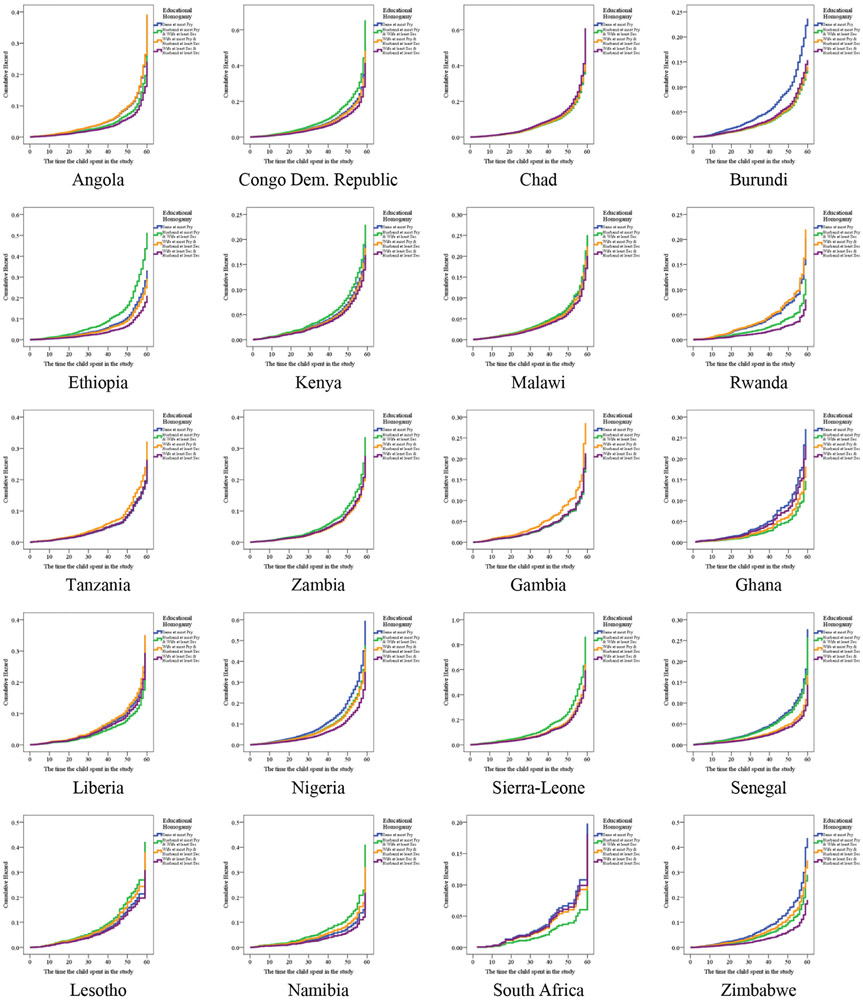
Pattern of Unadjusted hazard ratio of the relationship between joint parental education and under-five mortality in sub-Saharan African countries.

The pattern of hazard ratio of U5M across the categories of PEH is shown in Fig. 3. The pattern consistently observed across countries was, U5M was higher among children whose both parents had at most primary education than their counterparts whose both parents had attained secondary education or more. An important finding is that U5M was lower in couples where wives have at least secondary education and husbands have at most primary education than those households where wives have at most primary education and husbands have at least secondary education.

Variables included in the multivariate analysis and the identified predictors of U5M in each of the studied countries were presented in Table 2. Variables like age of mother at child’s birth, parity, sex, birth order, number of antenatal clinic visit, size of the child at birth, preceding birth interval and weight of the child at birth were most commonly used in the multivariate analysis for each country. These variables were used in at least 12 of the countries. However, as the main independent variable in this study, PEH was used in all the countries. Parental educational homogamy was identified as one of the predictors of U5M in only three countries, these are; Congo Democratic Republic, Gambia and Zimbabwe. Age of mother at the birth of the child was one of the identified predictors of U5M in Congo Democratic Republic, Rwanda, Tanzania and Nigeria. Children ever born was found to be a predictor of U5M in Angola, Congo Democratic Republic, Kenya, Rwanda and Nigeria. Countries where religion was found as U5M predictors are; Chad, Burundi, Zambia, Ghana and Liberia, whereas the household wealth was found as an U5M predictor in Angola, Burundi, Nigeria and Senegal.

The unadjusted and adjusted hazard ratio of the relationship between PEH and U5M are presented in Table 3 and further shown in Figs. 3a and b. In the table, the pattern of the relationship between these two variables barely changes from what was presented in the earlier part of this paper (Fig. 1). The bivariate logistic regression model showed that there was a significant relationship between PEH and U5M in 11 of the 21 studied countries. In Angola, the hazard ratio of U5M was 1.60 (C. I = 1.21–2.10, *p*<0.01) times and 1.63 (C. I = 1.20–2.20, *p*<0.01) higher among children whose both parents had at most primary education and where the mothers had lower education than fathers respectively compared to children of parents who have at least secondary education. This pattern exists in Burundi, Congo Democratic Republic, Rwanda, Nigeria, Namibia, and Uganda. In Rwanda, relative to under-five children who were born by parents with at least secondary education, the risk of under-five mortality was more in households where both parents had at most primary education 1.60 (C. I = 1.21–2.09, *p*<0.01), or where wife had at least secondary education and husband at most primary 1.64 (C. I = 1.11–2.43, *p*<0.05) or wife had at most primary and husband had at least secondary education 1.42 (C. I = 1.03–1.94, *p*<0.05).

Parental educational homogamy was identified as a predictor of U5M in Congo Democratic Republic, Gambia and Zimbabwe. In Congo Democratic Republic (CDR), being born by mothers who have at least secondary education and father having at most primary education 1.83 (C. I = 1.04–3.21, *p*<0.05) predisposes such children to higher risk of dying before reaching age five years compared to their counterparts who both parents have at least secondary education. The pattern exhibited in CDR was observed in Gambia and Zimbabwe. In Gambia, under-five children of parents who have at most primary education were 7.69 (C. I = 1.03–11.57, *p*<0.05) more likely to experience mortality than those whose parents had at least secondary education.

**Fig. 4.**
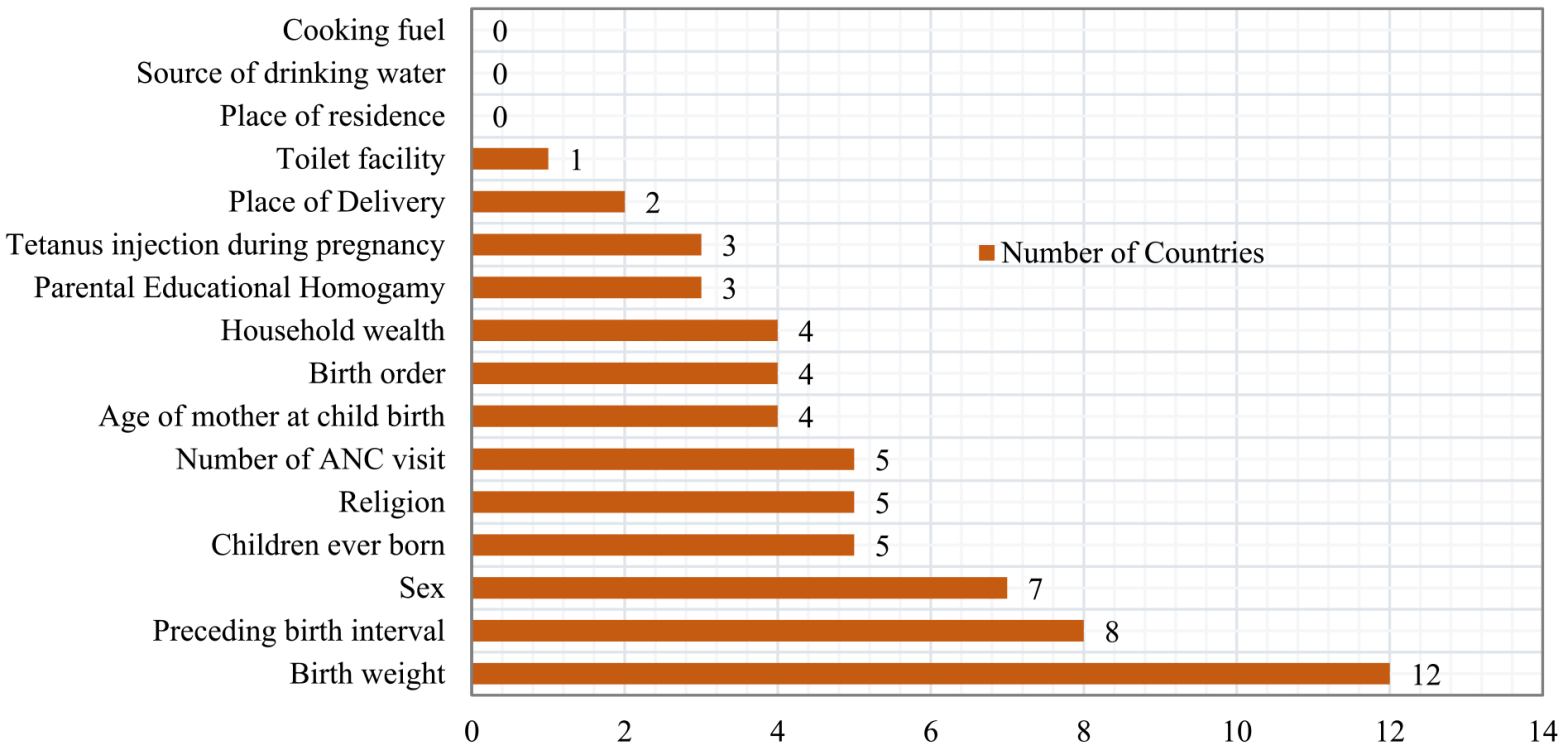
Distribution of variables according to the number of occurrence as U5M predictors in the 21 countries in Sub-Saharan Africa.

The distribution of variables according to the number of occurrence as U5M predictors was presented in Fig. 4. The data showed that birth-weight was found to be the most predictor of U5M across the 21 studied countries as its appears as a predictor in 12 countries. In Burundi for instance, the likelihood of U5M was 2.29(C. I = 1.62–3.23, *p*<0.05) times higher among the children who weigh <2.5 kg than those who weigh 3.5 kg and above. Preceding birth interval, sex of the child, children ever born and religion was an established predictor of U5M in 8, 7, 5 and 5 countries respectively. In almost all the countries where preceding birth interval was identified as U5M predictor, the U5M risk was least and highest experienced where women left 36 to 59 months’ and <24 months’ interval before the birth of the analyzed child. Surprisingly, place of residence, source of drinking water and cooking fuel were not being identified as U5M in any of the countries.

**Fig. 5.**
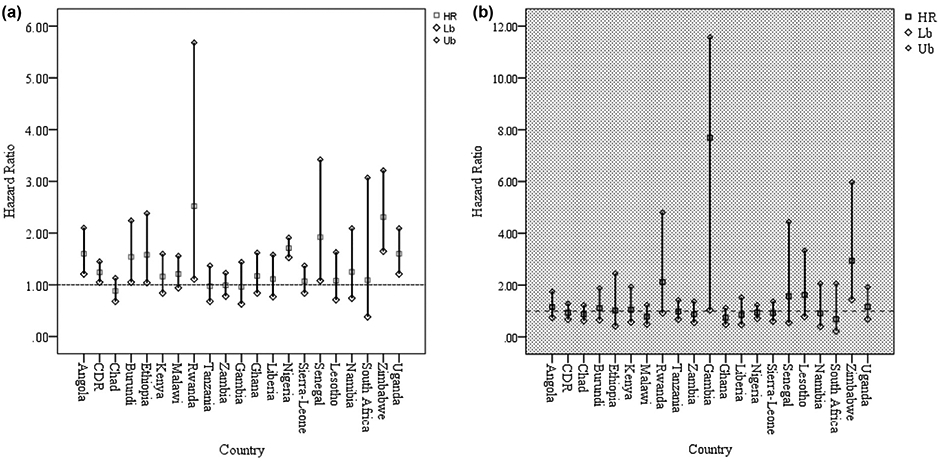
**(a)** Unadjusted Hazard Ratio for situations where Both husband and wife have at most primary education compared to where both husband and wife have at least secondary education. **(b)** Adjusted Hazard Ratio for situations where Both husband and wife have at most primary education compared to where both husband and wife have at least secondary education.

Fig. 5a and b shows the forest plot of the hazard ratio and 95% confidence interval of the comparison of hazard ratio of U5M in the extremes of PEH, where both parents have at most primary education on one hand (poorly educated) and at least secondary education on the other hand (good education). In the 11 countries where a significant relationship (unadjusted model) was established, the hazard ratio of U5M was higher among parents with at most primary education than children of parents with at least secondary education. A similar pattern was observed among the countries where PEH was identified as U5M predictor.

**Fig. 6.**
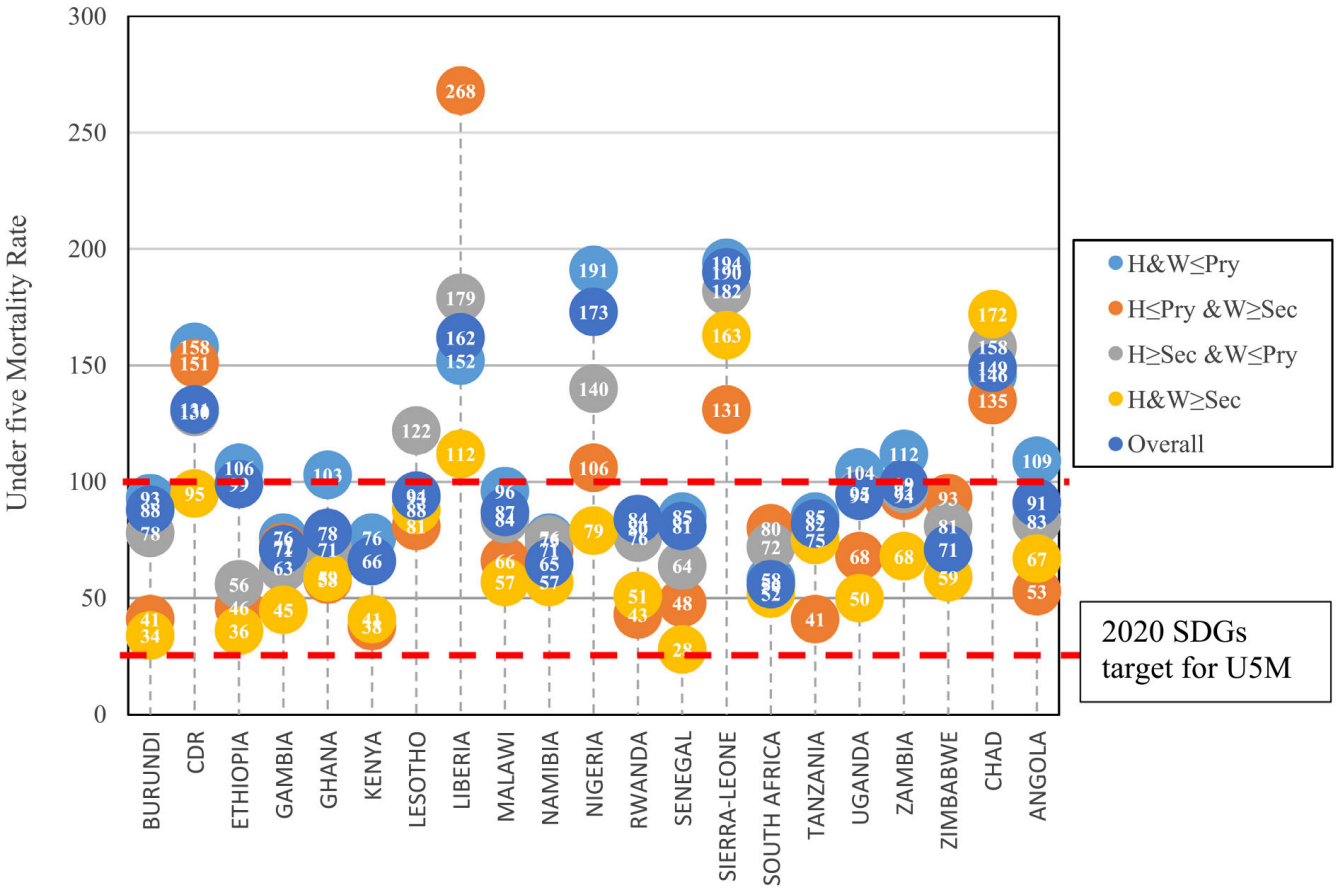
Estimated Under-five Mortality Rates by Country according to Educational Homogamy.

The estimated U5M rate per 1000 live birth in each of the categories of PEH across all the countries was presented in Fig. 6. The data show that in all the studied countries, U5M rate was higher among the children of parents with at most primary education than that of parents who had at least secondary education. This situation was also observed among children with parents where husbands were more educated than their wives. The U5M rate among parents who have joint education of at most primary education was 93/1000 in Burundi, 158/1000 in CDR, 106/1000 in Ethiopia, 76/1000 in Gambia, 103/1000 in Ghana, and 76/1000 in Kenya. This was higher than the U5M rate estimated for children of parents who had at least secondary education in Burundi (34/1000), CDR (95/1000), Ethiopia (36/1000), Gambia (45/1000), Ghana (59/1000), and Kenya (41/1000). It is noteworthy that U5M rate was lower among children of parents where the wives were more educated than husbands than where both parents had at least secondary education in 5 of the 21 countries. In 17 of the countries, the U5M rate was lower among children of parents where wives have at least secondary education and husband have at most primary education than families where wives have at most primary education and husbands have at least secondary education. In all the studied countries, U5M rate ranges from 56 per 1000 live born in South Africa to 190 per 1000 live born in Sierra-Leone.

## Discussion

Homogamy otherwise known as assortative mating has received considerable attention in literature but educational homogamy is a new dimension in social research [30]. Research on homogamy focused mainly on the cultural similarities of individuals with few issues addressed about educational homogamy [31,32]. The current study provides a broader overview of the relationship between PEH and U5M in SSA. It is a known fact that marriage partnership formation is often based on comparability in the level of education [12,13,30]. Habitually, when a couple have the same level of education, there is likelihood that their decision on daily activities will be similar, but it is not known in SSA whether PEH has influence or not on U5M. High childhood mortality in most SSA countries [21] has sustained low level of life expectancy in the region compared to other world sub-regions. As part of the efforts to reduce U5M in SSA, understanding the relationship between PEH and U5M is essential.

In this study, significant relationship was established between PEH and U5M in 11 out of the 21 countries studied but PEH was only identified as a predictor of U5M in Congo Democratic Republic, Gambia and Zimbabwe. An important finding is that U5M was least experienced in families where both husband and wife have not less than secondary education. The estimate of U5M rate obtained from the use of demographic method also supported this result. This outcome is expected and corroborated findings from previous studies where maternal’s and father’s education were used as independently predictors of U5M [18,24,33,34]. In most countries in SSA today, being employed, access to a job that attracts better remuneration and health-promoting benefits including health insurance coverage for all family members is a function of the individual’s level of education. Education influences preferences for child health and family size [35,36]. Conversely, the less educated people are more likely to be engaged in high-risk occupations that attract low income and benefits. From a diverse perspective, economic hardships at the family level as a result of poor education can be detrimental to the health of under-five children who are the most vulnerable member in the family. The job uncertainty, low earnings, unmet health needs, inability to pay utility bills for medical costs and poor housing conditions associated with low income and less education can make under-five children in the family to be more susceptible to diseases and infections. Access to health care has an influence on receipt of preventive services and care for illnesses, thus, poor access to health care has an implication on under-five survival [37]. Contrariwise, families that earn higher incomes have the tendency to easily acquire nutritious food, pay for health services and transportation to seek health care [37].

In 14 of the studied countries, U5M was lower in households where the wife is more educated than husbands compared to where the husband is more educated than the wife. This finding is in agreement with those from earlier studies which pointed at the importance of maternal education to child survival rather than paternal education [7,38]. Under-five children except for a very few exemptions stay with their mothers as their immediate care-needs are provided by their mothers while fathers may not be physically present. The strong causal relationship between maternal education and child survival has been widely explored in the literature [6,7,38,39]. Mother’s education may influence under-five mortality through different pathways. In African tradition, mothers provide primary care at the family unit while husbands ensure that daily expenses are covered by them. If a woman has good education and empowered, her earning will augment the husband’s earnings for the sustenance of the family needs. Principally, mothers acquisition of good education leads to better income, human, sociocultural capital, enhances the acquisition and utilization of health knowledge including health services. These indices are associated with improved child survival [6,7]. Husband’s education is also important, but this depends on their availability to respond to the family needs when required.

In all the studied countries, U5M rate ranges from 56 per 1000 live birth in South Africa to 190 per 1000 live born in Sierra-Leone. The U5M rate was found to be above 100 per 1000 live birth in Congo Democratic Republic, Liberia, Nigeria, Sierra-Leone and Chad. The pattern of U5M rate obtained in this study was similar to what was reported in previous studies in SSA [21,34]. Under-five mortality of at least 100 per 100 0 live birth in five of the studied countries was an indication of poor health service delivery and poverty level in the region. With regards to the achievement of 2030 SDGs agenda of achieving a reduction in U5M rate to 25 per 1000 live births [4], none of the studied countries had achieved this agenda and only South Africa is close to the target. However, in this study there is enough evidence to show that families where both husband and wife have at least secondary education will achieve the target. Also, there is a high possibility that the target can be achieved in families where the wife has a better education than husbands.

## Conclusion

The study revealed that PEH can influence the survival of their under-five children, but there is little evidence to sup-port this claim amidst other confounding variables. Under-five mortality was least in families where couples have at least secondary education and highest where couples have not more than primary education. Also, U5M was lower in families where wives are more educated than their husbands than vice versa. Therefore, it may be concluded that female literacy is pertinent to under-five survival in SSA.

## Recommendations

The findings from this study call for further research on the pathways through which the association between PEH and U5M develops. Ensuring that individuals particularly women have at least secondary education in accordance with the goal 4 of SDGs before marriage will facilitate a reduction in U5M in SSA. Strategic family educational programs on U5M reduction should be urgently instituted among couples with not more than primary education in countries such as Congo Democratic Republic, Liberia, Nigeria, Sierra-Leone and Chad where U5M rate was found to be above 100 per 1000 live birth. The high rate of U5M in SSA calls for initiatives aim at significantly reduce the level.

## Strengths and limitations of this study

The large nationally representative data from 21 different countries selected across sub-Saharan Africa added credence to our findings in terms of generalizability. The data used for this study were collected by USAID in collaboration with local agency from individual country is an evidence of good data quality. Under-five mortality estimates that are based on ret-rospective birth histories particularly age and age at deaths of children who died are often subject to misreporting errors which may distort the data quality. Inaccurate information on age at death may misrepresent the age pattern of mortality. However, the data originators ensured minimization of such errors at the point of data collection. Another important limitation is that the data for each country were not collected at the same time and as such, the timing of mortality will vary across the studied countries. Moreover, the quality of education as provided by the educational institutions in each country may be different. Therefore, the readers should be cautious in comparing the under-five mortality estimates among the countries.

## Figures and Tables

**Table 1:** Distribution of Under-five Mortality by Country according to background Characteristics.

Variables	Angola		CDR		Chad		Burundi		Ethiopia		Kenya		Malawi	
	%*d*	Total	%*d*	Total	%*d*	Total	%*d*	Total	%*d*	Total	%*d*	Total	%*d*	Total
**Total**	4.5	9376	7.1	15,456	8.3	16,176	4.8	11,569	5.5	9667	3.8	8239	4.2	13,719
**Residence**	**								*				***	
Urban	4.0	5076	6.6	4379	8.2	3289	3.9	1798	3.2	1696	3.7	2666	3.3	2173
Rural	5.2	4300	7.3	11,077	8.3	12,887	5.0	9771	6.0	7971	3.8	5573	4.3	11,546
**AMCB**	**		*		**		**						*	
< 20	6.2	1982	8.1	2629	9.4	3543	5.4	1016	6.8	1446	4.4	1220	5.5	3154
20–24	4.1	2583	7.2	4092	7.3	4607	5.9	3021	5.5	2839	3.6	2589	3.8	3992
25–34	3.9	3527	6.1	6508	8.1	6303	4.3	5614	5.2	4220	3.6	3474	3.4	5112
35+	4.7	1284	8.3	2227	9.2	1723	4.3	1918	5.0	1162	4.1	956	5.1	1461
**Parity**	*		**				***		***		**		**	
1	3.1	794	5.7	1357	5.6	1223	2.9	1045	3.8	1233	2.3	1001	3.1	2338
2–3	4.1	3382	6.2	4951	7.9	4675	5.0	4310	5.3	3122	3.5	3321	4.4	5716
4–5	3.4	1462	7.3	2330	7.0	2304	4.4	1686	5.8	1318	3.7	1251	3.6	1919
6+	5.6	3738	7.8	6818	9.3	7974	5.1	4528	6.1	3994	4.7	2666	4.8	3746
**Sex**	**				**		***		**		***		***	
Male	5.1	4696	7.3	7668	9.0	8211	5.2	5837	6.3	4976	4.2	4170	4.6	6894
Female	4.0	4680	6.9	7788	7.5	7965	4.4	5732	4.7	4691	3.3	4069	3.8	6825
**Birth Order**	*		*		*								*	
First Birth	6.1	1548	8.2	2596	9.3	2342	5.5	1987	6.2	1883	4.0	1609	5.9	3329
2–3	3.5	3418	6.2	4969	7.5	4770	4.9	4078	4.9	3065	3.5	3188	3.4	5214
4–5	3.8	2338	6.0	3847	7.1	4056	4.0	2857	5.5	2276	3.3	1860	3.1	3178
5+	5.9	2072	8.4	4044	9.5	5008	5.0	2647	5.7	2443	4.7	1582	5.1	1998
**Religion**			**		*		***		***					
Christianity	4.5	8715	6.9	14,709	10.2	4568	4.7	10,919	4.6	4473	3.7	6509	4.2	11,856
Islam	3.2	31			7.5	11,005	4.5	396	6.3	5018	4.2	1483	3.9	1781
Others	4.6	630	10.2	442	8.6	479	8.7	254	5.1	176	3.7	246	1.2	82
**HH Wealth**	*		**		***		*		*					
Poor	5.2	4845	7.0	7649	9.1	6263	6.6	4664	6.4	5283	3.9	4582	4.6	5846
Middle	4.9	2331	8.2	3329	8.0	3481	3.5	2297	4.9	1345	4.0	1350	4.2	2814
Rich	2.5	2200	6.3	4478	7.7	6432	3.6	4608	4.2	3039	3.4	2307	3.7	5059
**SDW**	**		*						***					
Unimproved	4.5	7523	7.6	10,579	8.0	7279	4.5	5998	6.2	3850	3.9	3244	5.0	1920
Improved	4.7	1772	5.9	4637	8.5	8731	5.1	5471	5.1	5703	3.8	4845	4.0	11,707
**Toilet F**			*				***		*					
Unimproved	5.3	4234	7.7	9827	8.4	14,179	4.9	11,099	5.9	8032	4.1	4840	4.2	2380
Improved	3.9	5142	6.0	5418	7.4	1782	2.3	437	3.3	1521	3.4	3253	4.2	11,247
**Cooking Fuel**	**				***				***					
Biomas	5.1	5455	7.1	15,064	8.4	15,611	4.8	11,515	5.7	9054	3.9	7558	4.2	13,471
Clean fuel	3.7	3840	5.2	193	5.6	396	0.0	21	2.4	499	2.6	538	4.5	156
**NANCV**			*						*		*		**	
None	3.6	1205	7.8	1185	5.3	4054	9.7	31	5.3	2255	6.1	310	6.4	171
1–3	3.0	1138	4.2	3827	5.1	2817	2.9	3647	2.6	1921	2.1	2244	2.5	5013
4+	2.4	3296	4.2	4176	4.7	2765	2.8	3816	2.2	2328	2.2	3216	2.6	5515
**PBI**	*		*		*		*		*		*		*	
0–23	7.5	2100	11.4	3520	12.9	4280	8.3	1793	9.0	1981	5.8	1301	7.1	1187
24–35	3.4	3059	6.1	5068	7.9	5312	4.2	3801	5.4	2533	3.4	2319	4.1	2739
36–59	2.5	1974	4.0	3373	3.6	3544	3.5	3295	2.7	2324	3.0	1863	2.8	4402
60+	2.6	695	3.7	899	3.6	698	3.3	693	3.9	946	3.1	1147	2.9	2062
**Birth Weight**	*		*				*		*		*		*	
< 2.5	5.6	468	10.9	597	6.2	128	9.4	746	3.1	192	6.6	288	4.7	1250
2.5–3.49	2.1	2141	6.0	4830	8.1	578	4.2	5492	2.0	817	2.5	2501	3.0	6205
3.5+	5.2	6767	7.1	9967	8.1	15,304	4.7	5331	5.9	8658	4.0	5415	5.2	6264
**TIDP**			**						*		***		***	
None	3.3	1616	5.7	2564	5.2	4158	2.8	3140	4.5	2930	3.5	719	3.5	1273
1+	2.5	3940	4.1	6569	4.8	5384	2.9	4349	2.5	3449	2.2	5009	2.5	9369
**POD**			*						*		***			
Home	4.8	5352	8.8	3877	8.1	13,199	6.0	1339	6.2	6621	4.1	3834	5.1	859
Health F	4.1	3905	6.2	11,363	8.0	2851	4.7	9751	3.9	2944	3.3	4300	4.1	12,667
Others	5.9	119	10.6	151	0.0	23	4.4	479	4.9	102	0.0	76	2.6	193
Variables	Rwanda		Tanzania		Zambia		Gambia		Ghana		Liberia		Nigeria	
	%*d*	Total	%*d*	Total	%*d*	Total	%*d*	Total	%*d*	Total	%*d*	Total	%*d*	Total
Total	3.4	6256	4.4	8276	4.8	10,230	3.4	7100	4.0	4796	5.9	5618	8.5	28,674
**Residence**													*	
Urban	2.4	1361	5.3	1790	4.9	3604	3.8	2285	4.2	1874	5.9	1623	5.9	9336
Rural	3.6	4895	4.2	6486	4.8	6626	3.1	4815	3.8	2922	6.0	3995	9.7	19,338
**AMCB**	**		*		*						**		*	
<20	6.0	366	6.6	1428	6.7	1796	4.1	1109	3.5	423	8.5	942	10.9	4495
20–24	2.9	1660	3.6	2139	4.9	2740	3.2	1889	3.2	1133	5.7	1411	8.1	7552
25–34	3.1	3275	3.7	3346	4.1	4307	3.0	3121	4.3	2338	5.6	2343	7.6	12,509
35+	4.1	955	5.3	1363	4.3	1387	3.8	981	4.3	902	4.6	922	9.2	4118
**Parity**	*								**				*	
1	2.1	1007	3.2	1071	3.5	967	3.5	888	2.0	601	4.0	501	6.1	3022
2–3	3.1	2839	4.7	3022	5.0	3656	3.1	2480	3.6	1923	5.8	1873	7.4	9798
4–5	2.6	844	3.8	1083	5.4	1512	3.9	945	3.4	764	7.0	804	8.4	4091
6+	5.2	1566	4.7	3100	4.7	4095	3.4	2787	5.4	1508	6.1	2440	10.0	11,763
**Sex**			***										*	
Male	3.7	3171	5.0	4216	5.1	5183	3.4	3640	4.1	2510	6.3	2851	9.1	14,561
Female	3.0	3085	3.8	4060	4.5	5047	3.3	3460	3.8	2286	5.6	2767	7.9	14,113
**Birth Order**	***		***		*		***						*	
First Birth	3.9	1608	5.8	1732	7.2	1719	4.5	1434	4.1	937	7.2	829	9.3	5314
2–3	2.5	2499	3.9	2789	4.4	3495	2.7	2332	3.4	1836	5.7	1853	7.2	9333
4–5	3.4	1231	3.8	1839	4.2	2531	3.4	1731	4.1	1232	6.3	1488	7.5	6795
5+	4.8	918	4.5	1916	4.3	2485	3.2	1603	4.9	791	5.1	1448	10.6	7232
**Religion**					*				***		*		*	
Christianity	3.4	6093	n.i	n.i	4.7	10,074	4.3	92	3.5	3342	5.3	4660	6.8	11,022
Islam	2.4	126	n.i	n.i	12.8	39	3.3	6997	5.5	1046	10.0	742	9.6	17,222
Others	3.4	29	n.i	n.i	15.7	89	n.i	n.i	3.7	408	5.2	210	8.2	282
**HH Wealth**													*	
Poor	3.9	2687	4.3	3712	5.0	4945	3.0	3613	4.0	2625	6.1	3648	11.1	13,358
Middle	3.2	1216	4.4	1598	5.1	2308	3.6	1549	4.0	827	6.1	1114	7.1	5531
Rich	2.8	2353	4.6	2966	4.3	2977	3.8	1938	3.9	1344	5.1	856	5.7	9785
**SDW**							***						*	
Unimproved	3.4	1715	4.3	3304	4.8	4234	4.5	880	3.8	1455	6.2	2116	9.5	11,800
Improved	3.3	4470	4.4	4594	4.8	5756	3.2	6131	4.1	3242	5.7	3385	7.8	16,593
**Toilet F**													*	
Unimproved	3.5	1782	4.3	5713	4.6	6334	3.3	3361	4.2	1998	5.9	3992	9.3	14,494
Improved	3.2	4397	4.7	2185	5.1	3648	3.4	3651	3.9	2699	5.9	1513	7.6	13,918
**Cooking Fuel**													*	
Biomas	3.3	6170	4.4	7734	4.9	9273	3.4	6975	4.0	3979	5.9	5500	9.2	23,503
Clean fuel	0.0	15	3.7	164	3.4	726	2.6	38	3.9	718	0.0	4	5.1	4928
**NANCV**					***		*						*	
None	6.5	31	3.1	97	7.1	99	28.6	28	3.9	102	6.3	142	6.9	6216
1–3	2.2	2498	2.4	2758	2.8	2953	2.1	982	1.7	343	5.1	759	5.7	2265
4+	2.1	2134	2.9	2764	2.4	3894	2.2	3717	2.3	3050	3.7	2979	4.5	9969
**PBI**					*		*		**		*		*	
0–23	3.9	719	4.9	1401	6.8	1375	6.1	788	5.5	474	10.6	859	13.2	5400
24–35	3.7	1457	3.9	2334	4.4	3291	2.6	2436	4.6	1170	5.0	1610	8.2	9192
36–59	2.8	1625	3.6	1867	3.2	2910	2.5	1784	4.1	1400	4.9	1537	5.3	6648
60+	2.5	847	4.0	942	4.0	935	2.7	658	1.8	815	3.4	783	5.8	2120
**Birth Weight**	*		**		*		*		*		*		*	
<2.5	7.3	273	9.1	242	7.1	506	5.1	393	3.5	198	4.0	100	5.5	308
2.5–3.49	2.6	2759	3.9	2719	3.3	3946	1.6	2314	1.4	1725	2.1	632	2.9	2340
3.5+	3.6	3220	4.5	5314	5.4	5741	3.9	4344	5.5	2870	6.4	4872	8.6	25,526
**TIDP**					**		**				***		*	
None	2.8	985	3.1	1579	3.7	1366	4.0	454	2.8	389	6.1	413	6.4	7277
1+	2.0	3665	2.5	4017	2.4	5529	2.0	4232	2.1	3076	3.8	3418	4.6	10,990
**POD**	*												*	
Home	7.0	457	4.0	3096	5.3	3252	3.0	3108	4.1	1520	6.6	2767	9.3	18,086
Health Facility	3.0	5704	4.6	4983	4.4	6849	3.5	3940	3.9	3258	5.1	2800	6.1	10,270
Others	6.4	94	6.1	197	3.3	92	4.5	22	0.0	17	8.1	37	5.9	34
Variables	Seirra-Leone		Senegal		Lesotho		Namibia		South Africa		Zimbabwe		Uganda	
	%*d*	Total	%*d*	Total	%*d*	Total	%*d*	Total	%*d*	Total	%*d*	Total	%*d*	Total
**Total**	9.8	9678	4.1	10,173	6.6	2377	4.2	2318	3.4	1377	4.6	4959	4.8	12,434
**Residence**											**		***	
Urban	9.9	2617	3.6	3201	6.5	567	3.5	1062	3.6	834	3.3	1838	3.9	2094
Rural	9.7	7061	4.3	6972	6.6	1810	4.9	1256	3.1	543	5.4	3121	5.0	10,340
**AMCB**											**		*	
<20	11.5	1637	4.9	1550	7.0	515	4.1	292	5.4	112	6.8	881	6.0	2399
20–24	10.0	2412	3.4	2517	6.4	793	5.0	540	2.3	298	4.2	1364	3.8	3544
25–34	9.1	4229	3.9	4482	6.4	829	3.3	1034	3.1	749	4.0	2151	4.3	4994
35+	9.6	1400	4.6	1624	6.7	240	5.5	452	5.0	218	4.4	563	6.7	1497
**CEB**	***		***		**		**		***		**		*	
1	9.1	1100	2.9	1334	3.5	687	2.8	356	2.3	221	2.3	860	4.5	1289
2–3	8.8	3426	4.3	3525	7.8	1192	3.2	1032	2.6	815	4.7	2494	4.0	4501
4–5	7.4	1572	3.6	1448	9.0	200	5.8	347	6.0	183	5.7	755	3.8	1730
6+	12.0	3580	4.5	3866	7.0	298	6.0	583	6.3	158	5.9	850	5.9	4914
**Sex**	***		***										**	
Male	10.4	4845	4.5	5248	6.6	1175	4.2	1128	3.6	717	5.1	2446	5.4	6279
Female	9.1	4833	3.6	4925	6.5	1202	4.3	1190	3.2	660	4.1	2513	4.1	6155
**Birth Order**	*		***										*	
First Birth	11.4	1724	4.8	2065	6.3	874	3.2	498	3.4	298	5.6	1240	5.8	2363
2–3	8.3	3366	3.6	3366	6.9	1050	4.0	1000	2.6	784	4.3	2326	3.6	4208
4–5	9.1	2576	3.5	2411	6.6	305	5.3	495	5.8	225	4.5	1047	4.6	2824
5+	11.8	2012	4.7	2331	6.1	148	4.9	325	5.7	70	4.0	346	5.8	3039
**Religion**			**											
Christianity	9.9	1659	1.9	216	6.6	2314	4.4	2024	n.i	n.i	4.7	4627	4.8	10,694
Islam	9.7	7972	4.1	9955	0.0	4	n.i	n.i	n.i	n.i	7.1	14	4.6	1572
Others	11.1	27	50.0	2	6.8	59	2.4	287	n.i	n.i	2.8	318	7.1	168
**HH Wealth**			***											
Poor	9.4	4434	4.4	5929	5.9	1120	5.2	1011	4.5	605	5.8	1932	5.2	6188
Middle	10.1	2022	4.2	2126	6.4	467	3.6	506	3.3	301	5.7	757	4.4	2411
Rich	10.2	3222	3.0	2118	7.6	790	3.4	801	2.1	471	3.2	2270	4.4	3835
**SDW**													***	
Unimproved	9.9	4448	4.6	2934	5.9	471	3.8	316	5.6	125	5.0	1010	5.5	2860
Improved	9.7	5182	3.9	6882	6.4	1699	4.2	1942	3.2	1193	4.5	3727	4.5	9232
**Toilet F**			***				***				*			
Unimproved	9.8	5360	4.7	4450	6.1	833	4.9	1378	4.7	403	6.1	1758	1.0	97
Improved	9.8	4277	3.6	5366	6.4	1337	2.9	882	2.8	915	3.7	2979	4.6	3136
**Cooking Fuel**					***		***				*			
Biomas	9.8	9631	4.2	8707	6.2	1480	4.9	1482	4.5	222	5.5	3184	4.7	12,044
Clean fuel	0.0	8	3.4	1109	6.4	690	2.8	778	3.2	1096	2.8	1553	2.1	48
**NANCV**	*		***		*		***				**		*	
None	14.8	149	4.2	212	10.6	66	3.7	82	3.6	56	7.8	217	11.9	135
1–3	8.9	618	3.4	2995	6.0	383	4.7	212	4.8	145	2.2	673	4.4	3019
4+	6.7	6087	2.7	3864	4.0	1514	2.9	1493	2.3	955	1.9	3014	2.7	4915
**PBI**	*		*								***		*	
0–23	16.8	1291	7.6	1347	10.0	180	7.2	291	4.2	95	6.7	435	6.5	2500
24–35	10.8	2799	3.2	3225	6.8	368	4.5	440	5.3	188	4.6	870	4.0	3846
36–59	6.2	2610	2.9	2620	6.2	486	3.8	525	2.7	329	3.1	1380	3.2	2767
60+	5.7	1254	3.7	916	6.0	469	3.7	564	3.0	467	4.6	1034	5.2	958
**Birth Weight**	*		*		*		**		*		*		*	
<2.5	7.5	268	5.6	608	12.6	183	6.4	202	2.7	147	7.4	326	6.7	714
2.5–3.49	3.9	2817	2.0	3283	4.7	1256	2.7	1099	1.1	715	2.7	2558	3.2	4018
3.5+	11.9	6462	5.0	6282	7.7	936	5.0	1001	6.8	515	6.6	2074	5.4	7702
**TIDP**					*						**		**	
None	9.6	218	3.8	843	8.4	357	3.6	692	3.6	304	4.6	584	5.2	1171
1+	6.8	6500	2.9	6112	3.7	1553	3.0	1007	2.7	698	1.9	3250	3.2	6879
**POD**	**										*			
Home	10.3	4319	4.7	2708	7.5	576	4.4	386	1.7	59	8.0	882	5.5	3354
Health Facility	8.8	5235	3.8	7341	6.2	1790	4.1	1909	3.5	1312	3.7	3948	4.5	8876
Others	20.0	25	5.6	124	0.0	9	0.0	13	0.0	6	7.8	129	5.9	204

AMCB: Age of mother at child’s birth; HH: Household; SDW: Source of Drinking water; F: Facility; NANCV: Number of ANC Visit; PBI: Preceding Birth Interval; TIDP: Tetanus Injection During Pregnancy; POD: Place of Delivery;%d: Percentage death

* *p*<0.001

** *p*<0.01

*** *p*<0.005.

n.i: Not included.

**Table 2:** Variables included in the multivariate analysis and the identified predictors of under-five mortality in sub-Saharan African countries.

Country	PEH	Place of Residence	AMCB	CEB	Sex	Birth Order	Religion	HH Wealth	SDW	Toilet Facility	Cooking Fuel	NANCV	PBI	Birth Weight	TIDP	POD
**Angola**	#	#	#	#†	#†	#†		#†	#		#		#†	#†		
**CDR**	#†		#†	# †		#	#	#	#	#†		#†	#†	#	#	#†
**Chad**	#		#		#†	#†	#†	#			#		#†			
**Burundi**	#		#	#	#†		#†	#†		#			#	#†		
**Ethiopia**	#	#		#	#		#	#	#	#	#	#	#	#	#	#
**Kenya**	#			# †	#							#	#	#	#	#
**Malawi**	#	#	#	#	#†	#						#†	#	#	#	
**Rwanda**	#		#†	#†		#								#†		#†
**Tanzania**	#		#†		#†	#								#		
**Zambia**	#		#			#	#†					#†	#†	#†	#†	
**Gambia**	#†					#			#			#†	#†	#†	#†	
**Ghana**	#			#			#†						#†	#†		
**Liberia**	#		#				#†						#	#†	#	
**Nigeria**	#	#	#†	#†	#†	#†	#	#†	#	#	#	#	#	#†	#	#
**Sierra-Leone**	#			#	#	#†						#	#†	#†		#
**Senegal**	#			#	#	#	#	#†		#		#	#	#†		
**Lesotho**	#			#							#	#		#	#†	
**Namibia**	#			#						#	#	#		#		
**South Africa**	#			#										#†		
**Zimbabwe**	#†	#	#	#						#	#	#	#	#	#	#
**Uganda**	#	#	#	#	#†	#			#			#†	#†	#†	#	
**N**_c_		6	12	16	11	12	9	7	6	8	7	13	16	20	11	7

AMCB: Age of mother at child’s birth; HH: Household; SDW: Source of Drinking water; NANCV: Number of ANC Visit; PBI: Preceding Birth Interval; TIDP: Tetanus Injection During Pregnancy; POD: Place of Delivery; #: Variables included in the model for multivariate analysis

† Identified predictors of under-five mortality (significant at *p*<0.05); N_c_ : Number of countries in which the association was established.

**Table 3:** Unadjusted and adjusted hazard ratio of the relationship between educational homogamy and under-five mortality in sub-Saharan African countries.

Country	*Unadjusted Hazard Ratio (95% C.I)*				*Adjusted Hazard Ratio (95% C.I)*			
	*H ≤ P&W ≤ P*	*H ≤ P&W ≥ S*	*H ≥ S&W ≤ P*	R.C	*H ≤ P&W ≤ P*	*H ≤ P&W ≥ S*	*H ≥ S&W ≤ P*	R.C
**Angola**	1.60(1.21–2.10)**	1.22(0.55–2.65)	1.63(1.20–2.20)**	1	1.15(0.75–1.75)	1.16(0.45–2.99)	1.32(0.87–1.98)	1
**CDR**	1.24(1.05–1.45)**	1.58(1.14–2.18)**	1.17(0.99–1.36)	1	0.93(0.67–1.28)	1.83(1.04–3.21)***	1.09(0.80–1.47)	1
**Chad**	0.88(0.68–1.13)	0.86(0.54–1.37)	0.90(0.67–1.21)	1	0.87(0.62–1.22)	1.08(0.62–1.89)	0.79(0.55–1.14)	1
**Burundi**	1.54(1.05–2.24)***	0.88(0.44–1.77)	0.92(0.50–1.70)	1	1.11(0.65–1.88)	0.67(0.26–1.69)	0.94(0.45–1.93)	1
**Ethiopia**	1.58(1.04–2.38)***	2.44(1.26–4.71)**	1.39(0.84–2.28)	1	1.02(0.42–2.45)	1.46(0.30–7.16)	0.72(0.25–2.02)	1
**Kenya**	1.16(0.84–1.60)	1.36(0.83–2.22)	1.09(0.72–1.64)	1	1.05(0.57–1.94)	1.84(0.83–4.05)	1.22(0.60–2.42)	1
**Malawi**	1.21(0.94–1.56)	1.25(0.81–1.90)	1.11(0.82–1.50)	1	0.78(0.49–1.23)	0.92(0.44–1.90)	0.72(0.43–1.23)	1
**Rwanda**	2.52(1.11–5.68)***	1.51(0.50–4.49)	2.73(1.01–7.38)***	1	2.12(0.92–4.80)	1.41(0.47–4.20)	2.38(0.87–6.50)	1
**Tanzania**	0.97(0.68–1.37)	1.01(0.59–1.72)	1.22(0.77–1.93)	1	0.98(0.68–1.42)	0.94(0.54–1.61)	1.10(0.68–1.76)	1
**Zambia**	0.99(0.78–1.23)	1.22(0.82–1.80)	0.95(0.73–1.23)	1	0.87(0.55–1.37)	0.91(0.42–1.97)	0.74(0.45–1.22)	1
**Gambia**	0.96(0.63–1.44)	0.97(0.52–1.79)	1.34(0.82–2.18)	1	7.69(1.04–11.57)***	5.21(0.53–9.42)	13.9(1.82–25.9)***	1
**Ghana**	1.17(0.84–1.62)	0.64(0.30–1.33)	0.78(0.50–1.21)	1	0.74(0.48–1.12)	0.50(0.21–1.18)	0.64(0.39–1.06)	1
**Liberia**	1.11(0.77–1.58)	0.84(0.37–1.88)	1.19(0.83–1.71)	1	0.85(0.47–1.52)	0.62(0.14–2.71)	1.11(0.63–1.96)	1
**Nigeria**	1.71(1.53–1.91)*	1.37(1.11–1.68)**	1.32(1.14–1.52)*	1	0.94(0.71–1.23)	1.15(0.78–1.67)	0.96(0.72–1.26)	1
**Sierra-Leone**	1.07(0.84–1.37)	1.45(1.01–2.10)***	1.08(0.81–1.42)	1	0.92(0.61–1.37)	1.48(0.80–2.72)	0.95(0.60–1.49)	1
**Senegal**	1.92(1.08–3.42)***	1.79(0.89–3.58)	1.15(0.56–2.36)	1	1.57(0.55–4.44)	0.84(0.20–3.38)	1.42(0.44–4.46)	1
**Lesotho**	1.08(0.71–1.63)	1.37(0.86–2.17)	1.24(0.66–2.30)	1	1.62(0.78–3.33)	1.96(0.98–3.90)	0.88(0.28–2.73)	1
**Namibia**	1.25(0.74–2.09)	1.89(1.09–3.23)***	1.48(0.82–2.64)	1	0.91(0.40–2.06)	1.03(0.42–2.50)	0.88(0.37–2.09)	1
**South Africa**	1.09(0.38–3.07)	0.61(0.18–1.97)	0.93(0.28–3.03)	1	0.68(0.22–2.06)	0.59(0.17–1.93)	0.73(0.22–2.39)	1
**Zimbabwe**	2.31(1.65–3.21)*	1.53(0.95–2.45)	1.84(1.31–2.57)*	1	2.94(1.44–5.97)***	0.99(0.33–2.94)	1.22(0.59–2.53)	1
**Uganda**	1.60(1.21–2.09)**	1.64(1.11–2.43)***	1.42(1.03–1.94)***	1	1.16(0.69–1.92)	1.45(0.72–2.89)	1.17(0.67–2.04)	1

* *p*<0.001

** *p*<0.01

*** *p*<0.005; *H ≤ P&W ≤ P*: Husband and wife have attained at most primary level of education; *H ≤ P&W ≥ S*: Husband has attained at most primary and wife has attained at least secondary level education; *H ≥ S&W ≤ P*: Husband has attained at least secondary and wife has attained at most primary level of education; R.C: Reference Category (Husband and wife have attained at least secondary education).

## References

[1] UNICEF, Data: Monitoring the Situation of Children and Women, United Nations Children Education Fund (UNICEF), 2018 https://data.unicef.org/topic/child-survival/under-five-mortality/USAID, 2018. Demographic and Health Survey. https://www.Dhs program.

[2] Alkema L, New JR, Global estimation of child mortality using a Bayesian B-spline bias reduction method’, Ann. Appl. Stat 8 (4) (2014) 2122–2149 Available at http://arxiv.org/abs/1309.1602.

[3] WHO, Children: Reducing Mortality, World Health Organisation, 2018 https://www.who.int/news-room/fact-sheets/detail/children-reducing-mortality.

[4] UN General Assembly. Transforming our world: the 2030 agenda for sustainable development, 21 10 2015, A/RES/70/1, available at: https://www.refworld.org/docid [accessed 16 April 2019].

[5] Liu L, Hill K, Oza S, Hogan D, Chu Y, Cousens S, Mathers C, Stanton C, Lawn J, Robert BE, Levels and causes of mortality under age five years, in: Black RE, Laxminarayan R, Temmerman M, (Eds.) editors, Reproductive, Maternal, Newborn, and Child Health: Disease Control Priorities, 2, The Int. Bank for Reconstruction and Development / The World Bank. Chapter 4, WashingtonDC, 2016. 27227226

[6] Akinyemi JO, Adebowale AS, Bamgboye EA, Ayeni O, Child survival dynamics in Nigeria: is the 2006 child health policy target met? Nigeria J. Health Sci 15 (2015) 18–26.

[7] Adebowale AS, Morakinyo OM, Ana GR, Housing materials as predictors of under-five mortality in Nigeria: evidence from 2013 demographic and health survey, BMC Pediatr. 17 (2017) 30. 28103828 10.1186/s12887-016-0742-3 PMC5248529

[8] Smith-Greenaway E, Maternal reading skills and child mortality in Nigeria: a reassessment of why education matters, Demography 50 (5) (2013) 1551–1561. 23592326 10.1007/s13524-013-0209-1 PMC3786132

[9] Huber S, Educational homogamy lowers the odds of reproductive failure, PLoS ONE 6 (7) (2011) e22330 10.1371/journal.pone.0022330. 21818310 PMC3144227

[10] Becker GS, A Treatise on the Family, 1st ed., Harvard University Press, Cambridge, Mass., 1981.

[11] Schwartz C, Mare R, Trends in educational assortative marriage from 1940 to 2003, Demography 42 (4) (2005) 621–646. 16463914 10.1353/dem.2005.0036

[12] Mare R, Educational Assortative Mating in Two Generations, Dep. Sociology, Univ. California, Los Angeles, 2008 Working Paper http://opr.princeton.edu/seminars/papers/RobertMare.pdf .

[13] Siow A, Testing becker’s theory of positive assortative matching, J. Labor Econ 33 (2) (2015) 409–441.

[14] UNESCO. Fact Sheet Sub-Saharan Africa, Strong foundations: early childhood care and education, The UN Educational, Scientific and Cultural Organization, 2017 https://en.unesco.org/gem-report/sites/gem-report/files/fact_sheet_ssa.pdf.

[15] Fernández R, Rogerson R, Sorting and long-run inequality, Q. J. Econ 116 (2001) 1305–1341.

[16] Akombi BJ, Agho KE, Hall JJ, Wali N, Renzaho AMN, Merom D, Stunting, wasting and underweight in sub-Saharan Africa: a systematic review, Int. J. Environ. Res. Public Health 14 (8) (2017) 1pii: E863, doi:10.3390/ijerph14080863. 28788108 PMC5580567

[17] Bain LE, Awah PK, Geraldine N, Kindong NP, Sigal Y, Bernard N, Tanjeko AT, Malnutrition in sub-Saharan Africa: burden, causes and prospects, Pan. Afr. Med. J 15 (2013) 120, doi:10.11604/pamj.2013.15.120.2535. 24255726 PMC3830470

[18] Aristidem RB, Susuman AS, Women’s education and health inequalities in under-five mortality in selected sub-saharan african countries, 1990–2015, PLoS ONE 11 (7) (2016) e0159186, doi:10.1371/journal.pone.0159186. 27442118 PMC4956109

[19] Frost MB, Forste R, Haas DW, Maternal education and child nutritional status in Bolivia: finding the links, Soc. Sci. Med 60 (2) (2005) 395–407 j. socscimed. 10.1016/0000-0001-7903-5035. 15522494

[20] Sonalde D, Soumya A, Maternal education and child health: is there a strong causal relationship? Demography 35 (1) (1998) 71–81. 9512911

[21] Population Reference Bureau. World population data sheet. A special focus on human needs and resources. 2018. http://www.prb.org/pdf16/prb-wpds2016-web-2016.pdf.

[22] USAID. The Demographic and Health Surveys (DHS) Program. United States agency for international development. 2018. https://www.dhsprogram.com/.

[23] Grépin KA, Bharadwaj P, Maternal education and child mortality in Zimbabwe, J. Health Econ 44 (2015) 97–117. 26569469 10.1016/j.jhealeco.2015.08.003

[24] Morakinyo OM, Fagbamigbe AF, Neonatal, infant and under-five mortalities in nigeria: an examination of trends and drivers (2003-2013), PLoS ONE 12 (8) (2017) e0182990, doi:10.1371/journal.pone.0182990. 28793340 PMC5549979

[25] Mosley WH, Chen LC. An analytical framework for the study of child survival in developing countries population development review. 1984, 10:25–45. PMC2572391 12756980

[26] Garenne M, Enéas G, Under-five Mortality Trends in Africa: Reconstruction from Demographic Sample Surveys, ORC Macro, Calverton, Maryland, USA, 2005 DHS Working Papers No. 26 Available athttp://dhsprogram.com/pubs/pdf/WP26/WP26.pdf.

[27] Klein JP, Moeschberger ML, Survival Analysis Techniques For Censored and Truncated Data, 2nd Ed., Springer-Verlag New York Berlin Heidelberg, 2003 ISBN 0-387-95399-X.

[28] Moultrie TA, Dorrington RE, Hill AG, Hill K, Timæus I, Zaba B, Tools For Demographic Estimation, International Union for the Scientific Study of Population. Demographic estimation, Paris, 2013 iussp.org.

[29] United Nations. Manual X: indirect techniques for demographic estimation (United Nations publication, Sales No. E.83.X111.2). 1983.

[30] Schwartz CR, Mare RD, “Trends in educational assortative marriage from 1940 to 2003, Demography 42 (4) (2005) 621–646. 16463914 10.1353/dem.2005.0036

[31] Marco HD, Van L, Ineke M, A historical community approach to social homogamy in the past, Hist. Fam 24 (1) (2019) 1–14 1570532, doi:10.1080/1081602X.

[32] Matthijs K, Intermarriage and homogamy: causes, patterns, trends, Annu. Rev. Sociol 24 (1988) 395–421. 10.1146/annurev.soc.24.1.395 12321971

[33] Yaya S, Uthman OA, Okonofua F, Bishwajit G, Decomposing the rural-urban gap in the factors of under-five mortality in sub-Saharan Africa? Evidence from 35 countries, BMC Public Health 19 (1) (2019) 616 Epub 2019 May 21. 10.1186/s12889-019-6940-9 PMC6528236 31113395

[34] Yaya S, Bishwajit G, Okonofua F, Uthman OA, Under five mortality patterns and associated maternal risk factors in sub-Saharan Africa: a multi-country analysis, PLoS ONE 13 (10) (2018) e0205977, doi:10.1371/journal.pone.0205977. 30359408 PMC6201907

[35] Prickett KC, Augustine MJ Jennifer M, Maternal education and investments in children’s health, J. Marriage Fam 78 (1) (2016) 7–25, doi:10.1111/jomf.12253. 26778853 PMC4712746

[36] Adebowale AS, Palamuleni ME, Childbearing dynamics among married women of reproductive age in Nigeria: re-affirming the role of education, Afr. Popul. Stud 27 (2) (2014) 302–318.

[37] Chokshi DA, Income, poverty, and health inequality, JAMA 319 (13) (2018) 1312–1313, doi:10.1001/jama.2018.2521. 29614168

[38] Caldwell JC, McDonald P, “Influence of maternal education on infant and child mortality: levels and causes, Health Policy Educ. 2 (1982) 251–267. 10256648 10.1016/0165-2281(82)90012-1

[39] Cleland JG, Ginnekin JJV, “Maternal education and child survival in developing countries: the search for pathways of influence, Soc. Sci. Med 27 (1988) 1357–1368. 3070762 10.1016/0277-9536(88)90201-8

